# Time trends in post-polypectomy surveillance guideline adherence: analysis of over 90 000 colonoscopies with polypectomy

**DOI:** 10.1055/a-2701-7611

**Published:** 2025-10-10

**Authors:** Jasmin Zessner-Spitzenberg, Daniela Penz, Elisabeth Waldmann, Florian Bognar, Sarah Unger, Theresa Selinger, Alexandra Demschik, Michael Trauner, Monika Ferlitsch

**Affiliations:** 1 Internal Medicine III, Division of Gastroenterology and Hepatology, Medical University of Vienna, Vienna, Austria; 2 Quality Assurance Working Group, Austrian Society for Gastroenterology and Hepatology, Vienna, Austria; 3 Department of Gastroenterology, Endocrinology and Geriatrics, Klinik Floridsdorf, Vienna, Austria

## Abstract

**Background:**

Previous studies found poor adherence to guidelines on post-polypectomy care. We aimed to investigate adherence to the European Society of Gastrointestinal Endoscopy (ESGE) recommendations before and after publication of the updated guideline in 2020, stratified by polyp type.

**Methods:**

Participants undergoing screening documented in the Austrian Quality Certificate for Screening Colonoscopy program were included in the study. Piecewise linear regression with one breakpoint was applied to investigate trends in guideline adherence. A mixed-effects logistic regression model was fitted to investigate the associations of screening participant and endoscopist characteristics with guideline adherence.

**Results:**

Between August 2020 and November 2024, adherence to the post-polypectomy guideline increased by 49% (95%CI 19%–86%). The strongest improvement in surveillance interval adherence was observed for adenomas (38.4% in 2020 vs. 51.2% in 2024); adherence for serrated polyps only marginally improved (52.4% in 2020 vs. 54.3% in 2024). There was a positive association between endoscopists’ adenoma detection rate (ADR) and the probability of adherence to the ESGE guideline for surveillance of high risk polyps; however, the effect size was only modest (odds ratio 1.01, 95%CI 1.00–1.03).

**Conclusions:**

An improvement of up to 49% in ESGE guideline adherence was observed over 4 years after publication of the update, which was mostly driven by improved adherence to the surveillance intervals for adenomas. Endoscopists with higher ADRs were more likely to assign correct follow-up intervals.

## Abstract (de)

**Graphical Abstract:**

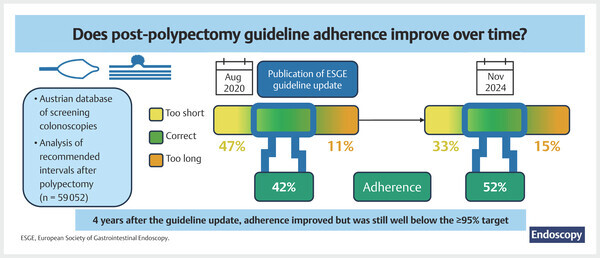

## Introduction

Individuals with high risk polyps have an elevated risk for colorectal cancer (CRC) compared with those without high risk features 1 2 3 4 . Surveillance colonoscopy aims to reduce the risk of subsequent CRC for these individuals, and involves a repeat colonoscopy after several months or 3 years, depending on the polyp findings and polypectomy technique 5 6 7 . In 2020, the European Society of Gastrointestinal Endoscopy (ESGE) updated the recommendations for these intervals, with high risk individuals defined as those with ≥5 adenomas, a serrated polyp/adenoma ≥10 mm, a serrated polyp with dysplasia, or an adenoma with high grade dysplasia 8 . These individuals should receive a written recommendation for follow-up colonoscopy in 3 years, while others can return to screening with colonoscopy in 10 years. Individuals who undergo piecemeal resection of large polyps should have a repeat colonoscopy in 6 months. The main changes in the ESGE guideline update were the introduction of high risk serrated polyps as a new entity requiring surveillance, and the exclusion of villous histology as a high risk feature in adenomas. Additionally, the multiplicity of adenomas requiring surveillance increased from 3 to 5 9 . For large polyps removed by piecemeal resection, the cutoff for short-term follow-up colonoscopy of 6 months was elevated from 10 mm to 20 mm 8 .

Adherence to post-polypectomy guidelines is notoriously poor and is consistently reported to be below the 95% target standard proposed by the ESGE in many countries 10 11 12 . Notably, interventions such as reminder letters sent to endoscopists are not sufficient to significantly improve guideline adherence 13 . Over- and under-use of surveillance colonoscopy might have detrimental effects: an interval that is too long might miss polyps that have recurred, and one that is too short exposes individuals to the unnecessary harms associated with colonoscopy. Nonadherence to interval recommendations adds to the colonoscopy demand and can impact the cost-effectiveness of colonoscopy procedures and screening programs 14 .

The introduction of the universal 10-mm cutoff for high risk polyps irrespective of histology and removal of villous growth pattern as a high risk criterion has simplified stratification. Little is known about whether this simplification has helped to improve guideline adherence or about the temporal trends in guideline adherence by polyp type since the introduction of the 2020 guideline update. The aim of this retrospective analysis was to assess adherence to the 2020 post-polypectomy guideline intervals, to reveal the trends in adherence before and after publication of the update, and to investigate whether guideline interval adherence differed by polyp histology (conventional adenoma or serrated polyp).

## Methods

### Study setting

Austria has an opportunistic primary colonoscopy screening program, where individuals ≥50 years irrespective of sex are eligible. Since 2007, the screening program has been accompanied by a nationwide quality assurance program, the Austrian Quality Certificate for Screening Colonoscopy. The aim of this voluntary program is to monitor screening colonoscopy performance and to provide feedback for endoscopists by giving access to biennial benchmarking reports. Participation in the program is not a requirement for reimbursement of screening colonoscopy. Endoscopists are required to upload data of screening colonoscopies to obtain certification; these include patient demographics, colonoscopy findings, and data on colonoscopy quality parameters such as bowel preparation and cecal intubation. The data are acquired through a standardized form, which can be accessed through the endoscopists’ electronic health record or an online browser-based application. The recommended follow-up interval in months is a non-mandatory field in this form. The uploaded data are audited once a year, when endoscopists are asked to share the written colonoscopy report of a random sample of the uploaded screening colonoscopies. One random sample is drawn from colonoscopies where a polyp was detected, and two random samples are drawn from negative colonoscopies. Only when data uploaded to the database align with the original colonoscopy report is the data audit passed. If endoscopists fail the audit, they are contacted and reminded of the program’s certification standards.

The period of certification spans 2 years, and endoscopists are required to apply for renewal of participation at the end of each cycle. To become re-certified, endoscopists need to have passed the two data audits and have uploaded a sufficient number of colonoscopies to ensure an adequate sample size for the benchmarking of key performance measures. With the biennial invitation for re-certification, a letter is sent to each endoscopist with information on current or previous guideline updates concerning screening colonoscopy. In the invitation letter at the end of 2021 for the re-certification period of 2022–2023, the guideline update for polypectomy, which was published by the ESGE in August 2020 and by the Austrian Society for Gastroenterology and Hepatology in December 2021, was included. The post-polypectomy recommendations of the national society are in line with the recommendations of the ESGE.

The study was approved by the Ethics Committee of the Medical University of Vienna (EK 1095/2025).

### Study population

Individuals undergoing screening colonoscopy and consenting to data transfer to the Austrian Quality Certificate for Screening Colonoscopy were included. We excluded screening participants who were diagnosed with CRC at colonoscopy, who had inadequate bowel preparation (poor or insufficient bowel preparation on the Aronchick scale), incomplete colonoscopies (as defined by not reaching the cecum), individuals without polyps at screening, colonoscopy reports without a recommendation for follow-up colonoscopy (either screening or surveillance recommendation), and those without complete polypectomy. Incomplete polypectomy was defined as a colonoscopy where not all polyps were removed. We also excluded individuals who received diagnoses other than colorectal polyps at screening colonoscopy. These exclusion criteria were applied to ensure that only high quality colonoscopies with complete polypectomy, to which the guidelines apply, were used for the analyses.

### Definition of variables and follow-up

#### Low risk screening participants

In line with the revised 2020 post-polypectomy ESGE guidelines, screening participants who had low risk findings were defined as those with 1–4 adenomas, polyps <10 mm, adenomas with low grade dysplasia, or serrated polyps without dysplasia.

#### High risk screening participants

High risk participants were defined as those with polyps ≥10 mm, an adenoma with high grade dysplasia, or a serrated polyp with dysplasia. Those with ≥5 adenomas were also considered high risk individuals, irrespective of the presence of high risk features in polyps.

#### Correct colonoscopy interval recommendation

The correct colonoscopy interval recommendation was defined by the colonoscopy finding. We considered a correct follow-up interval as “return to screening” for low risk findings (i.e. a repeat colonoscopy after 10 years [120 months]). The correct interval for high risk screening participants was defined as 36 months if high risk polyps were sized 1–19 mm. If polyps ≥20 mm were detected at colonoscopy, the correct interval was follow-up of 3–6 months. The ESGE also recommends this short interval for piecemeal polypectomy of nonpedunculated polyps ≥20 mm. However, only the type of snare used for polypectomy was recorded in the database, not whether piecemeal resection was performed. Therefore, follow-up recommendations for sessile or flat polyps ≥20 mm that underwent attempted complete polypectomy were defined as “correct” if 3–6 months of follow-up were recorded by the endoscopist. Follow-up of pedunculated polyps ≥20 mm was correct if assigned to 36 months.

### Statistical analysis

**Fig. 1:**
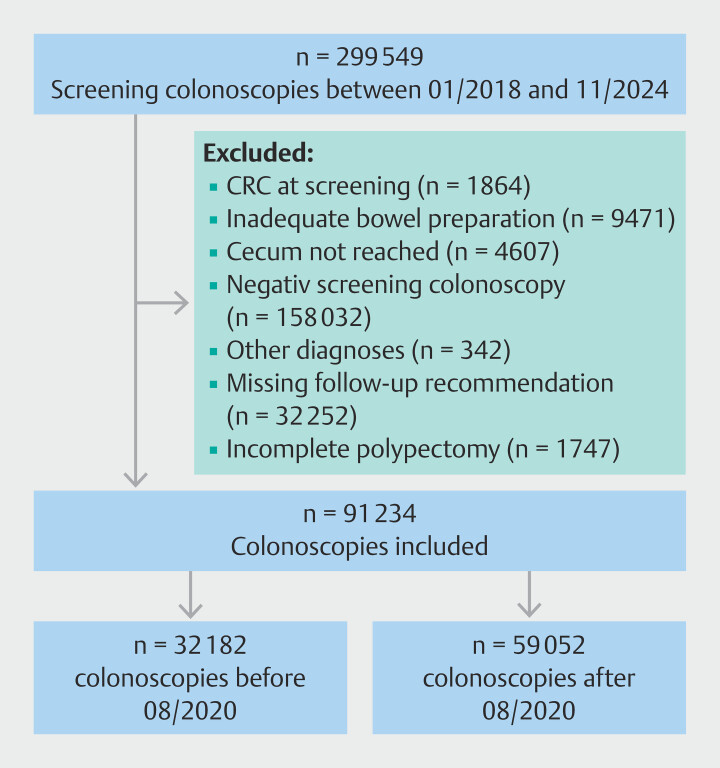
Flow chart of colonoscopies with polypectomy performed in 2018–2024. CRC, colorectal cancer.

Descriptive statistics of polyp findings were applied for colonoscopies performed after August 2020 by absolute and relative frequencies for categorical variables, and means with SDs and medians with interquartile ranges (Q1, Q3) for continuous variables. The relative frequency of correctly recommended surveillance intervals per year was calculated as the total number of colonoscopies in which participants with high risk polyps received the correct recommendation for high risk polyps (36 months) divided by all colonoscopies performed in that year overall and subgrouped by endoscopy specialty. A separate analysis of recommended follow-up was performed for low risk polyps and polyps ≥20 mm. As incomplete histological removal of polyps might trigger shorter follow-up interval recommendations, we performed a sensitivity analysis where the correct recommendation rate for high risk polyps was calculated only for histologically confirmed completely excised polyps. To investigate the time trends in surveillance interval recommendations, we performed a piecewise linear regression with one breakpoint where the dependent variable was frequency (%) of correct surveillance intervals per month since August 2020. This model was chosen to identify a potential inflection point in the recommendations over time. The appropriateness of model fit was assessed by visualizing the model’s residuals. Additionally, we investigated whether a more flexible model (spline transformation of time with two degrees of freedom) could yield a better fit to the data (see **Fig. 1s** in the online-only Supplementary Material).

We were interested in the effect of the year of colonoscopy on the probability of a correct surveillance interval. We fitted a mixed-effects logistic regression model with the year of colonoscopy adjusted for age and sex of the participants and specialty of the endoscopists. The estimates of this model are reported as odds ratios (OR) with 95%CIs. We chose the endoscopists performing the colonoscopies as a random effect to account for clustering between physicians. As there were only a few repeat colonoscopies per patient, we did not add a random effect for the patient level.

**Fig. 2:**
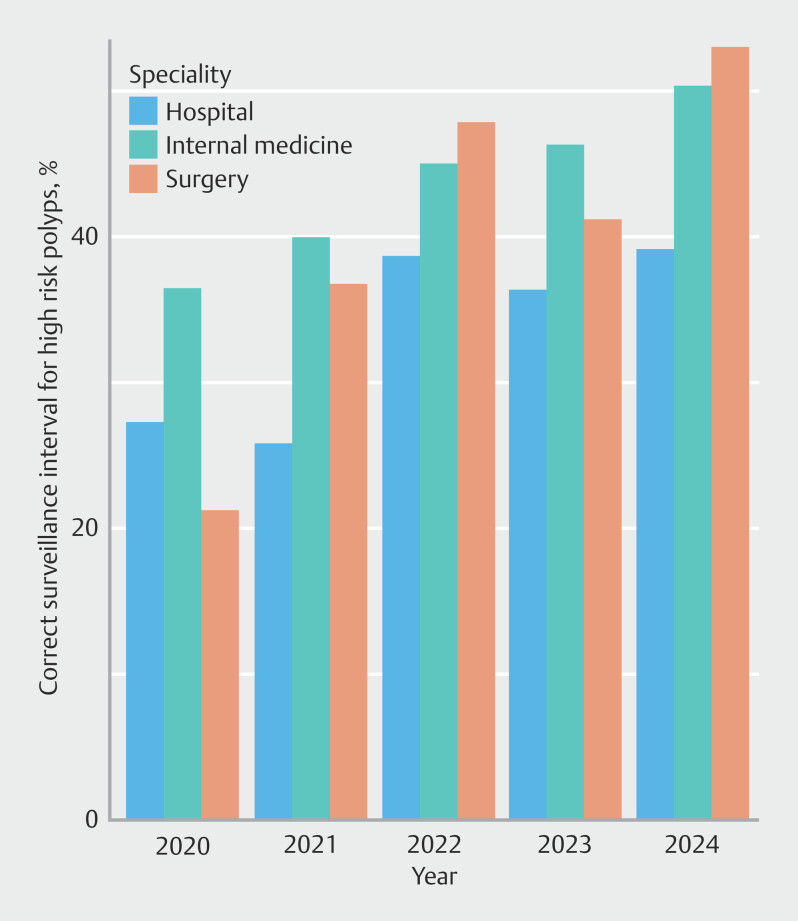
Trends in guideline adherence over time by specialty.

We calculated the endoscopists adenoma detection rate (ADR) as the sum of all detected adenomas up until the most recent colonoscopy divided by all colonoscopies performed in a dynamic manner 15 . This way of calculating differs from interval-based ADRs (i.e. monthly or yearly ADRs) in that they are updated at every single colonoscopy performed. The dynamic ADR therefore allows for fluctuations over time, capturing its variability. Another advantage of the dynamic ADR is that it only uses the data of detected adenomas up until the current time point and does not rely on data points “ahead in time” (which would be the case in interval-based ADR calculations) 15 . The dynamic ADR and the year of endoscopy were added to the regression model as fixed effects. The model structure is depicted in **Fig. 2s** .

We performed a sensitivity analysis where polyps ≥20 mm were excluded, as there were only limited data on the resection technique for these lesions. This could introduce bias due to a variability in the recommended follow-up interval.

*P* < 0.05 was considered significant for hypothesis testing according to year of endoscopy; the *P* value for trend was calculated with the mixed model. All analyses were performed with R version 4.4.2 with the packages segmented version 2.1–3 and lme4 version 1.1–35.5 (R Foundation for Statistical Computing, Vienna, Austria).

## Results

### Baseline characteristics

Of 91 234 colonoscopies with polypectomy in which polyps were detected ( Fig. 1 ), 59 052 were performed after 1 August 2020. Colonoscopies were performed by 342 endoscopists. Of these procedures, 9491 were performed in hospital endoscopy units, 18 624 by private practice surgeons, and 30 937 by private practice internal medicine specialists. Polyp removal with either hot or cold snare (n = 30 741) and/or forceps (n = 37 559) was performed. There were 6746 colonoscopies with high risk polyps requiring surveillance, of which 6325 were screening colonoscopies and 421 were surveillance colonoscopies. The median number of detected polyps was 2.0, and 9.1% of colonoscopies with detected polyps reported a size of >10 mm ( Table 1 ). Overall, the most advanced pathology was a serrated polyp in 22 579 colonoscopies, and adenomas were detected in 36 473 colonoscopies.

**Table:** **Table 1** Baseline characteristics of polypectomies performed after publication of the updated guideline for post-polypectomy surveillance by the European Society of Gastrointestinal Endoscopy (August 2020).

Variable	Colonoscopies ^1^ (N = 59 052)
Age	
Mean (SD)	63.7 (8.62)
Median (Q1, Q3)	63.0 (57.0, 70.0)
Sex, n (%)	
Female	26 618 (45.1)
Male	32 434 (54.9)
High risk polyp, n (%)	
Yes	6746 (11.4)
No	52 306 (88.6)
Specialty, n (%)	
Hospital	9491 (16.1)
Internal medicine	30 937 (52.4)
Surgery	18 624 (31.5)
Polyp size (mm), n (%)	
<5 mm	34 458 (58.4)
5–10 mm	19 183 (32.5)
11–19 mm	4553 (7.7)
≥20 mm	847 (1.4)
Missing	11 (0.02)
Shape, n (%)	
Flat	18 046 (30.6)
Pedunculated	5013 (8.5)
Sessile	35 982 (60.9)
Missing	11 (0.0)
Number of polyps	
Mean (SD)	2.2 (1.9)
Median (Q1, Q3)	2.0 (1.0, 3.0)
Missing	2 (0.02)
Adenoma count	
Mean (SD)	1.7 (1.3)
Median (Q1, Q3)	1.0 (1.0, 2.0)
Missing	16 994 (28.8)
Indication, n (%)	
Surveillance	4157 (7.0)
Screening	54 895 (93.0)
Q, quartile. ^1^ Colonoscopies with polypectomy performed from August 2020 to November 2024.	

### Interval recommendations for polyps requiring surveillance

The frequency of correctly assigned surveillance intervals in high risk individuals improved during the study period, with the strongest increase for adenomas ( Table 2 ). In 2020, 38.4% of participants with high risk adenomas and 52.4% of participants with high risk serrated polyps received the correct surveillance recommendation, which increased to 51.2% and 54.3% in 2024, respectively ( **Table 1s** ). Overall, 41.5% of follow-up recommendations were correct in 2020, and this increased after publication of the guideline update to 51.9% by November 2024, without a change in the median length of the surveillance recommendation (36 months). In 2020, 11.2% of surveillance interval recommendations were too long; this also increased to 15.4% by November 2024. However, the frequency of surveillance intervals that were too short decreased, from 47.4% in 2020 to 32.6% in 2024. There were 818 colonoscopies after August 2020 with polyps ≥20 mm, for which a correct follow-up recommendation of 6 months was given to 30.1% of participants in 2020 and 33.3% in November 2024 ( **Table 2s** ). An incorrect interval length of >6 months was assigned for 32.9% in 2020, increasing to 41.5% in 2024 for these polyps. Given that poorer bowel preparation might prompt endoscopists to recommend shorter follow-up intervals, we performed a stratified analysis for surveillance recommendations by bowel preparation degree ( **Table 3s** ). The proportion of correct surveillance interval recommendations was highest for individuals with excellent bowel preparation compared with good or adequate bowel preparation ( **Table 3s** ). The rate of correctly assigned surveillance intervals only marginally improved when histologically confirmed incompletely excised polyps were removed from the analysis (51.9% vs. 54.2%) ( **Table 4s** ).

**Table:** **Table 2** Median surveillance recommendations in months and their frequency in percent for high risk polyps ^1^ requiring surveillance according to the 2020 European Society of Gastrointestinal Endoscopy guideline for post-polypectomy surveillance.

	Median surveillance interval, months (% of surveillance interval assignments)				
	2020	2021	2022	2023	2024
Adherence to guideline recommendation					
Correct	36 (41.5)	36 (44.3)	36 (47.3)	36 (49.3)	36 (51.9)
Too long	60 (11.2)	60 (10.5)	60 (13.6)	60 (12.8)	60 (15.4)
Too short	12 (47.4)	12 (45.2)	12 (39.2)	12 (38.0)	12 (32.6)
^1^ High risk polyps are defined as adenomas ≥10 mm, adenomas with high grade dysplasia, ≥5 adenomas, serrated polyps ≥10 mm, or serrated polyps with dysplasia.					

### Interval recommendations for polyps not requiring surveillance

Only 1.6% of screening participants with low risk polyps received the correct interval recommendation for follow-up colonoscopy in 2020; this increased to 14.9% by November 2024. While 98.4% of low risk polyps were recommended intervals that were too short in 2020 (median 36 months) (Table 3), this proportion decreased to 85.1% (median 60 months) ( Table 3 ) by November 2024. After August 2020, there were 139 individuals with low risk polyps who previously had high risk polyps removed at colonoscopy. The median interval recommendation was 60 months in this group. In individuals with low risk polyps after previous removal of polyps ≥20 mm (n = 22), the median recommended interval was also 60 months.

**Table:** **Table 3** Median surveillance recommendations in months and their frequency in percent for low risk polyps ^1^ not requiring surveillance according to the 2020 European Society of Gastrointestinal Endoscopy guideline.

	Median surveillance interval, months (% of surveillance interval assignments)				
	2020	2021	2022	2023	2024
Adherence to guideline recommendation					
Correct	120 (1.6)	120 (4.0)	120 (7.3)	120 (11.3)	120 (14.9)
Too short	36 (98.4)	36 (95.9)	48 (92.7)	60 (88.7)	60 (85.1)
Too long	–	168 (0.1)	220 (0.1)	360 (0.1)	366 (0.1)
^1^ Low risk polyps are defined as adenomas or serrated polyps <10 mm, serrated polyps without dysplasia, adenomas with low grade dysplasia, or <5 adenomas.					

### Temporal trends in surveillance interval recommendations

Before the introduction of the updated ESGE post-polypectomy surveillance guideline, 34.7% of endoscopists adhered to the 2013 guideline recommendations for advanced adenomas in 2018; this rate remained stable throughout 2020 before publication of the updated guideline ( **Table 5s** ). After publication of the updated guideline in August 2020, adherence to the recommended intervals for high risk polyps gradually improved over time; however, the strongest positive slope was observed in the first years after publication ( Fig. 2 ). Each year after 2020 was associated with improved guideline adherence ( *P* trend <0.001), with the largest estimate observed for the year 2024 (OR 1.49, 95%CI 1.19–1.86) ( Table 4 ). In our sensitivity analysis, where polyps ≥20 mm were excluded, the estimates for the year of endoscopy were comparable ( **Table 6s** ).

**Table:** **Table 4** Mixed logistic regression model of the association of patient characteristics and endoscopist characteristics with guideline adherence.

Characteristic	OR	95%CI	P value	*P* trend
Year (vs. 2020)				<0.001
2021	1.15	0.92–1.43	0.20	
2022	1.35	1.08–1.68	0.008	
2023	1.39	1.12–1.74	0.003	
2024	1.49	1.19–1.86	<0.001	
ADR	1.01	1.00–1.03	0.06	
Specialty (vs. hospital)				
Internal medicine	1.31	0.95–1.81	0.10	
Surgery	0.82	0.57–1.19	0.30	
Sex (vs. female)				
Male	0.95	0.85–1.05	0.30	
Age	1.00	0.99–1.00	0.12	
ADR, adenoma detection rate; OR, odds ratio.				

### Association of endoscopist characteristics with guideline adherence

**Fig. 3:**
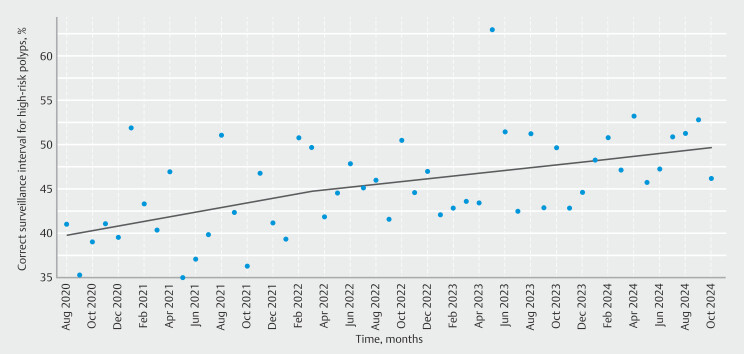
Trends in guideline recommendations since publication of the updated European Society of Gastrointestinal Endoscopy guideline for post-polypectomy surveillance (August 2020).

We found no significant association of the endoscopist specialty (OR for surgery 0.82, 95%CI 0.57–1.19; OR for internal medicine 1.31, 95%CI 0.95–1.81) or setting other than private practices compared with hospitals and adherence to the recommended guidelines ( Fig. 3 ). However, there was a positive association of endoscopist’s ADR with the probability of adhering to the ESGE guideline for surveillance of high risk polyps (OR 1.01, 95%CI 1.00–1.03).

## Discussion

In the present study, we observed a general improvement in adherence to the recommended guideline in a nationwide CRC screening program in Austria. However, the recommended intervals were only correct in 51.9% of individuals with high risk findings and 14.9% of individuals with low risk polyps at screening colonoscopy. Adherence to post-polypectomy guidelines was found to be poor in many previous studies 11 16 17 18 19 20 . The reasons for nonadherence are not entirely understood. Endoscopists who provide high quality colonoscopies and have a higher ADR are more likely to provide the correct recommendations to patients 13 . In our study, we found that the ADR, as a measure of high performing endoscopists, was positively associated with a higher probability of assigning a correct surveillance interval. However, the effect size of the estimate was only small, implying that various other factors contribute to the variability in surveillance recommendations.

A study from a tertiary academic center in the USA supports the notion that the endoscopists’ preferences rather than patient characteristics lead to incorrect interval recommendations. Nonadherence to the United States Multi-Society Task Force on Colorectal Cancer post-polypectomy guideline was more frequent for endoscopists who finished training more than 10 years ago or those who had a higher annual colonoscopy volume, while endoscopist sub-specialty, patient sex, or patient age showed no significant association 21 . Our data are in line with these findings, as we also found no significant association of endoscopy specialty or participant characteristics with guideline nonadherence. Another study from Italy found that high volume centers in the national screening program and the provision of written recommendations were associated with correct surveillance intervals 22 . Motives for shorter intervals than necessary might be attributable to overestimation of the time to polyp recurrence risk, lack of knowledge of the guideline recommendations, or financial interests 20 23 24 25 .

Schoen et al. found poor adherence to post-polypectomy intervals in community practice, and a high overuse of repeat colonoscopy in low risk individuals 25 . Our data from average risk individuals from a nationwide colonoscopy screening program confirm the disproportionally low adherence in low risk participants compared with high risk individuals, although the correct recommendations generally improved during the 4-year study period.

The improvements in guideline adherence in our study from 2020 to 2024 were observed in participants with polyps requiring surveillance (high risk findings) and polyps not requiring surveillance (low risk findings), but the strongest improvements were in the group of participants not requiring surveillance (1.6% in 2020 vs. 14.9% in 2024). Interestingly, the correct surveillance rate remained steady for serrated polyps but strongly improved for adenomas during the study period. Two factors might have contributed to this observation. On the one hand, there could be a rising awareness that villous adenomas or adenoma multiplicity are no longer high risk features. On the other hand, it has long been held that serrated polyps are innocuous lesions, prompting endoscopists to recommend longer follow-up intervals compared with the follow-up for adenomas 26 . Adherence to the surveillance intervals for advanced adenomas from the 2013 ESGE guideline was poorer in the control period (January 2018–August 2020), which might be explained by the more simplified guideline recommendations introduced in 2020 8 9 . The ESGE recommendations for surveillance of serrated polyps remained mainly unchanged in the guideline update of 2020. However, awareness of these kinds of polyps is still poor, which is why the adherence to the guideline for high risk serrated polyps experienced almost no improvement over time. This might be due to a lack of confidence in the guideline recommendations; most of the incorrectly assigned intervals were too short in this category. Although regular educational efforts such as seminars and courses were provided by the project lead of our quality assurance program, serrated polyp management remains only fair.

In general, we observed a reduction in surveillance overuse during the study period, which mostly stemmed from a reduction in shorter intervals for low risk findings. However, we also observed a small increase in surveillance recommendations that were too long for high risk polyps (11.2% in 2020 and 15.4% in 2024). The correct surveillance recommendations for polyps ≥20 mm remained relatively steady. The strongest improvements in guideline adherence over time were observed in the first years after publication of the update, but only significantly improved in the year 2024. A study from the Netherlands addressed adherence to the 2020 ESGE post-polypectomy guideline. Among a survey of 84 gastroenterologists, the authors found a median correct interval recommendation in 71% of answers. In contrast to our study, the correct interval rate was lower for serrated polyps 27 .

Several measures can be taken to improve guideline adherence. Quality improvement initiatives posting current guidelines where they are most visible (workstations, pocket cards) can help physician awareness 28 . To overcome the issue of incorrectly assigned recommendations by endoscopists, automatic reminders to primary care providers and patients can be distributed through electronic health records 29 . However, an important and well-established practice to ensure that surveillance is utilized is a written recommendation on the colonoscopy report 8 . Patients are often not aware of the need for surveillance after colonoscopy 30 .

Our study had a large sample size of over 90 000 high quality colonoscopies with polypectomy from a nationwide registry. Another strength of our study is that we analyzed the trends in post-polypectomy guideline adherence spanning several years, rather than adopting a cross-sectional design. Additionally, we were able to assess the temporal trends in guideline adherence for adenomas and serrated polyps separately. We only used high quality endoscopies, as recommendations for follow-up colonoscopy with poor bowel preparation or failed cecal intubation would have skewed the data toward shorter recommendations 31 . The data structure allowed for sensitivity analyses to identify potential biases toward surveillance intervals that were shorter than recommended, such as histologically confirmed incomplete removal of polyps or bowel preparation that was worse than “excellent.”

Limitations of this study include the lack of standardized polyp size evaluation, which is recommended by the ESGE. Polyp size measurement was left to the discretion of endoscopists. The lack of data on the resection technique used for lesions ≥20 mm is a limitation; only the type of snare is recorded, but not whether piecemeal resection was attempted. This information is crucial for the distinction between a 3-year and a 3–6-month follow-up interval. However, piecemeal resection is the most common practice for flat/sessile lesions ≥20 mm in Austria, which is why all flat/sessile polyps were assumed to be removed by piecemeal resection. Another limitation is that we cannot prove whether the electronically uploaded recommended interval was also stated on the written colonoscopy report. Endoscopists enter data personally in the electronic report form, which should be derived from the written report they provide their patients. Therefore, we can only assume that that the uploaded interval is in fact what endoscopists advised their patients. Another limitation is that we could not analyze the within-center variability of interval recommendations, owing to the database structure.

In conclusion, when societies publish guidelines for post-polypectomy surveillance intervals, a rise in adherence to the recommendations can be expected; in our study, however, only small increments in guideline adherence were observed over a 4-year period.

## Supplementary Material

Supplementary Material Zusatzmaterial
