# Supplementary material for: Time trends in post-polypectomy surveillance guideline adherence: analysis of over 90 000 colonoscopies with polypectomy
Source: Endoscopy. 2025 Oct 10;58(3):233–41. doi: 10.1055/a-2701-7611 (PMC13077560; doi:10.1055/a-2701-7611)

Supplementary material

Time trends in post-polypectomy surveillance guideline adherence: analysis of over 90 000 colonoscopies with polypectomy

Jasmin Zessner-Spitzenberg, Daniela Penz, Elisabeth Waldmann, Florian Bognar, Sarah Unger, Theresa Selinger, Alexandra Demschik, Michael Trauner, Monika Ferlitsch

Table 1s Median recommended interval in months for high risk serrated polyps and high risk adenomas.

Median interval in months (%) for high-risk serrated polyps	2020	2021	2022	2023	2024
correct	36 (52.43%)	36 (51.6%)	36 (52.49%)	36 (52.71%)	36 (54.25%)
too long	60 (11.65%)	60 (14.8%)	60 (19.27%)	60 (15.95%)	60 (15.34%)
too short	12 (35.92%)	12 (33.6%)	12 (28.24%)	12 (31.34%)	12 (30.41%)
**Median interval in months (%) for high-risk adenomas**	**2020**	**2021**	**2022**	**2023**	**2024**
correct	36 (38.44%)	36 (42.38%)	36 (45.74%)	36 (48.13%)	36 (51.17%)
too long	60 (11.02%)	60 (9.33%)	60 (11.96%)	60 (11.7%)	60 (15.47%)
too short	12 (50.54%)	12 (48.28%)	12 (42.3%)	12 (40.17%)	12 (33.36%)

Table 2s Median recommended interval for high risk polyps ≥20 mm.

Median interval in months (%) for high-risk polyps ≥20 mm	2020	2021	2022	2023	2024
correct	6 (30.14%)	6 (31.68%)	6 (32.98%)	6 (37.11%)	6 (33.33%)
too long	12 (32.88%)	12 (35.64%)	12 (41.36%)	12 (37.63%)	12 (41.52%)
too short	12 (36.99%)	12 (32.67%)	12 (25.65%)	12 (25.26%)	12 (25.15%)

Endoscopy | DOI 10.1055/a-2701-7611 | © The Author(s).

Table 3s Median recommended surveillance interval for high risk polyps in excellent, good, or fair bowel preparation.

**Median interval in months (%), excellent bowel preparation**	**2020**	**2021**	**2022**	**2023**	**2024**
correct	36 (50%)	36 (51.24%)	36 (55.72%)	36 (56.1%)	36 (59.23%)
too long	60 (12.84%)	60 (12.62%)	60 (15.66%)	60 (14.94%)	60 (16.07%)
too short	12 (37.16%)	12 (36.14%)	12 (28.62%)	12 (28.96%)	12 (24.7%)
**Median interval in months (%), good bowel preparation**	**2020**	**2021**	**2022**	**2023**	**2024**
correct	36 (34.13%)	36 (39.72%)	36 (40.3%)	36 (44.58%)	36 (48.58%)
too long	60 (9.13%)	60 (8.98%)	60 (12.65%)	60 (10.68%)	60 (13.99%)
too short	12 (56.73%)	12 (51.3%)	12 (47.05%)	12 (44.75%)	12 (37.42%)
**Median interval in months (%), fair bowel preparation**	**2020**	**2021**	**2022**	**2023**	**2024**
correct	36 (34.69%)	36 (36.08%)	36 (41.51%)	36 (39.31%)	36 (35.5%)
too long	60 (12.24%)	60 (8.23%)	60 (9.43%)	60 (11.56%)	60 (18.34%)
too short	12 (53.06%)	12 (55.7%)	12 (49.06%)	12 (49.13%)	12 (46.15%)

Table 4s Median recommended surveillance interval and relative frequency of interval recommendations in individuals with histologically proven complete excision of polyps.

**Median interval in months (%) for high-risk polyps**	**2020**	**2021**	**2022**	**2023**	**2024**
correct	36 (45.99%)	36 (47.2%)	36 (49.65%)	36 (50.47%)	36 (54.22%)
too short	60 (9.89%)	60 (9.83%)	60 (12.74%)	60 (13.58%)	60 (16.07%)
too long	12 (44.12%)	12 (42.97%)	12 (37.61%)	12 (35.94%)	12 (29.71%)

Table 5s Median recommended interval in months for advanced adenomas before the introduction of the updated polypectomy guideline by the ESGE.

**Median interval in months (%) for advanced adenomas**	**2018**	**2019**	**2020**
correct	36 (34.7%)	36 (34.39%)	36 (34.98%)
too long	36 (26.37%)	48 (26.63%)	36 (29.06%)
too short	12 (38.93%)	12 (38.99%)	12 (35.96%)

Endoscopy | DOI 10.1055/a-2701-7611 | © The Author(s).

Table 6s Sensitivity analysis. Mixed logistic regression model of the association of patient characteristics and endoscopist characteristics with guideline adherence of polyps 1 – 19 mm.

Characteristic	OR[1]	95% CI[1]	p-value
**year**			
2020	—	—	
2021	1.15	0.90, 1.46	0.3
2022	1.31	1.03, 1.67	0.027
2023	1.43	1.12, 1.82	0.004
2024	1.48	1.16, 1.89	0.002
**ADR**	1.01	1.00, 1.03	0.093
**specialty**			
hospital	—	—	
internal medicine	1.30	0.91, 1.87	0.15
surgery	0.82	0.54, 1.23	0.3
**sex**			
f	—	—	
m	0.91	0.81, 1.02	0.10
**age**	0.99	0.99, 1.00	0.058

[1] OR = Odds Ratio, CI = Confidence Interval

Thieme

Fig. 1s function of correctly assigned surveillance intervals and time with natural splines and two degrees of freedom.

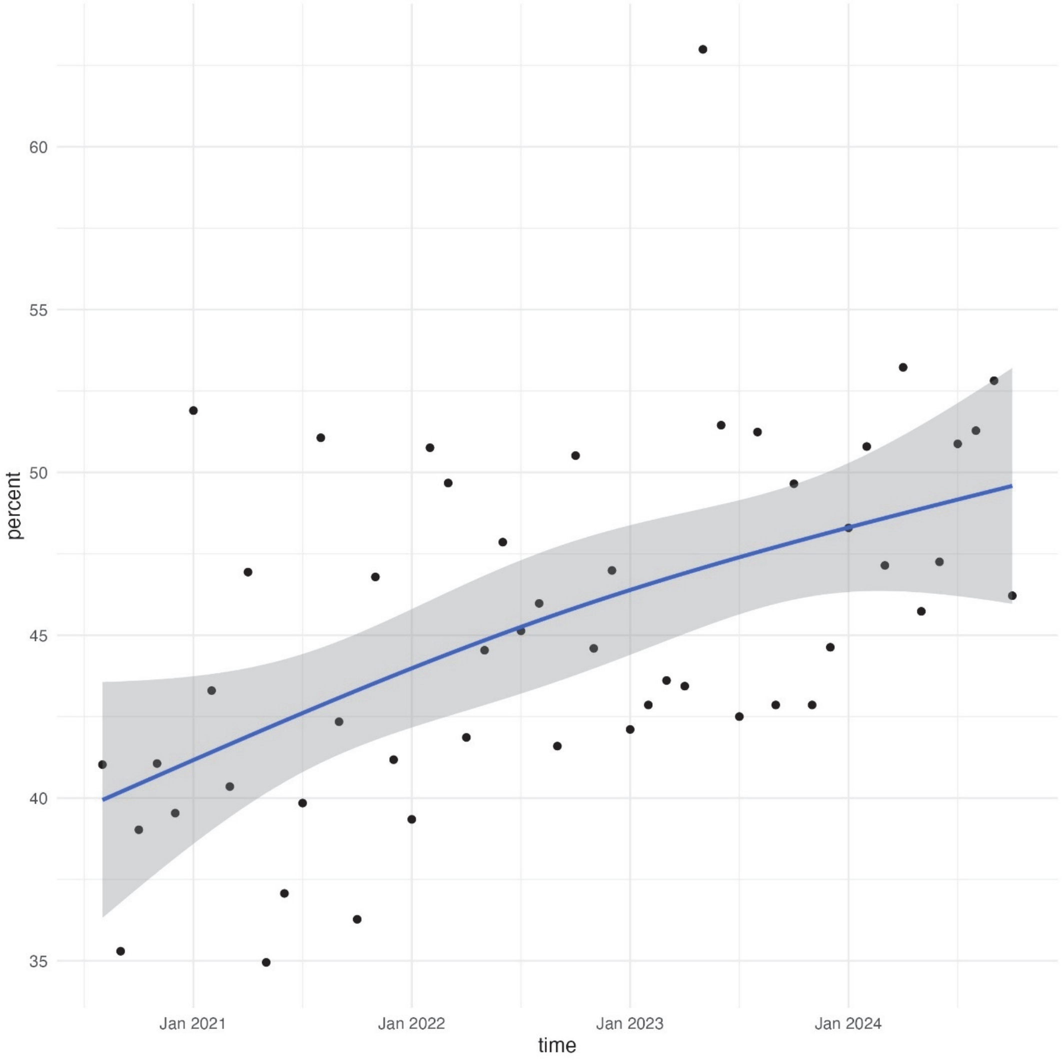

Fig. 2s Directed acyclic graph of the mixed model to assess the association of patient sex, age, endoscopist specialty, year of endoscopy, ADR (fixed effects) and endoscopists (random effect) with the probability of a correctly assigned surveillance interval.

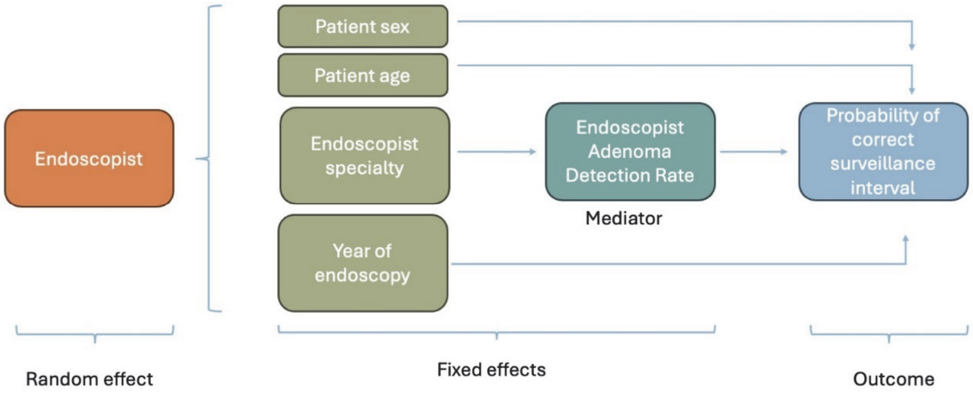

Supplement: Supplementary file 1 — Supplementary Material [file 10-1055-a-2701-7611_27018323.pdf]
